# Genital Tract Interleukin-8 but not Interleukin-1β or Interleukin-6 Concentration is Associated with Bacterial Vaginosis and Its Clearance in HIV-Infected and HIV-Uninfected Women

**DOI:** 10.1155/2007/92307

**Published:** 2007-10-11

**Authors:** Phyllis Losikoff, Raina Fichorova, Brad Snyder, Irma Rodriguez, Susan Cu-Uvin, Joseph Harwell, Kenneth H. Mayer

**Affiliations:** ^1^Department of Pediatrics, Division of Infectious Diseases, Warren Alpert Medical School, Brown University, Providence, RI 02912, USA; ^2^Hasbro Children's Hospital, Providence, RI 02903, USA; ^3^Laboratory of Genital Tract Biology, Brigham and Women's Hospital, Department of Obstetrics and Gynecology, Harvard Medical School, Boston, MA 02115, USA; ^4^Center for Statistical Sciences, Brown University, Providence, RI 02912, USA; ^5^Miriam Hospital, Providence, RI 02906, USA; ^6^Department of Medicine, Division of Infectious Diseases, Warren Alpert Medical School, Brown University, Providence, RI 02912, USA

## Abstract

Genital tract infections and cytokine perturbations are associated with increased HIV acquisition and transmission. We measured the relationship between bacterial vaginosis (BV) and concentrations of Interleukin-8 (IL-8), Interleukin-1β (IL-1β), and Interleukin-6 (IL-6) in cervicovaginal lavage (CVL) specimens collected longitudinally from 16 HIV-infected and 8 HIV-uninfected high-risk women. CVL samples were analyzed when women presented with BV, and at their next visit, after successful treatment, when BV was cleared. A subset of participants had cytokine levels evaluated at three consecutive clinic visits: before developing BV, at the time of BV diagnosis, and after clearing BV. Significantly higher IL-8, but not IL-1β or IL-6 levels were present when women had active BV compared to when BV was absent. Trends in cytokine levels were similar for HIV-infected and HIV-uninfected women. BV in these women was associated with significantly higher concentrations of genital tract IL-8 which decreased 2.4 fold when BV was cleared.

## 1. INTRODUCTION

Bacterial vaginosis (BV) is associated with higher risk of HIV acquisition [1]. In HIV-infected women, BV is associated with higher concentrations of HIV RNA in genital tract secretions [2, 3]. Thus, BV may increase a woman's risk of transmitting HIV infection to her sexual partner or infant. The exact mechanisms by which BV enhances HIV acquisition and viral shedding are not well understood. Recent studies suggest that BV's role in enhancing HIV transmission may be the result of both direct effects of pathogenic microorganisms on HIV expression, and indirect effects arising from BV-related alterations in the local mucosal immune environment by increasing the expression of proinflammatory cytokines [4, 5].

Proinflammatory cytokines such as Interleukin-1β (IL-1β) and Interleukin-6 (IL-6) directly induce HIV-1 replication through NF-*κ*B-mediated activation of HIV-1 long terminal repeat [6]. Interleukin-8 (IL-8), which recruits cells susceptible to HIV, directly enhances HIV replication in macrophages and T-lymphocytes [7]. A recent study of note by Narimatsu et al. demonstrated that IL-8 significantly increases susceptibility of cervical tissue to HIV infection [8]. Though BV is considered a noninflammatory disorder, several cross-sectional studies have reported higher genital tract concentrations of proinflammatory cytokines in women with BV compared to healthy controls [9–12]. Thus, it is possible that higher genital tract HIV viral loads in HIV-infected women and enhanced susceptibility to infection in seronegative women may be mediated directly by the effects of abnormal microflora on genital tract cytokine expression.

The scientific understanding of the range of “normal” genital tract cytokine concentrations is incomplete, partially because these concentrations vary widely, partly due to host factors including the local vaginal milieu and the genetic diversity of participants that cannot be controlled in cross-sectional studies [13]. Investigations of BV and genital tract cytokine concentrations have generally not included HIV-infected women, despite their higher prevalence of BV and the potential role BV could play in facilitating HIV transmission to sexual partners and offspring.

The aim of the present study was to compare levels of the cytokines IL-1β, IL-6, and IL-8 in HIV-infected and high-risk HIV-uninfected women in the presence and absence of BV. These specific cytokines were chosen because of their distinct roles in HIV-1 pathogenesis and their possible use as surrogate biomarkers for vaginal inflammation. The longitudinal study design eliminated individual variability when comparing inflammatory cytokines in the genital tract. The use of archived CVL specimens facilitated the selection of longitudinal samples from women with clear changes in BV status and avoidance of confounding factors that may impact the ability to assess the effects of BV on CVL cytokine concentrations.

## 2. METHODS

Participants were chosen from women enrolled in the HIV Epidemiological Research Study (HERS) in Providence Rhode Island [14]. The HERS cohort included HIV-infected and demographically matched high-risk controls who were evaluated every six months in four U S urban centers from 1994 to 2001. At each visit, a complete pelvic exam was performed that included collection of a cervicovaginal lavage (CVL) specimen and a vaginal swab. We selected archived samples from women who had been diagnosed with BV, treated, and had no evidence of BV at the next consecutive HERS visit, six months later. A subset of participants was evaluated at three consecutive visits at six month intervals. These women did not have BV at study entry which was defined as the “baseline” visit. They acquired BV while they were HERS participants and they were treated, which resulted in clearance of BV. Women who were pregnant or had concomitant infection with *Trichomonas vaginalis*, *Neisseria gonorrhea*, or *Chlamydia trachomatis* at any of the visits were not included in this study. Also excluded were women who reported douching, having intercourse, or inserting any intravaginal products into their vagina within the 48 hours before CVL samples were obtained. A microscopic exam of vaginal fluid for sperm was performed. Specimens from women who were menstruating, or if the practitioner could visualize blood at the cervical os or in the vagina, at the time of CVL collection at any visit, were not selected for inclusion in this study. Diagnostic testing methods for this cohort have been described in detail elsewhere [14]. Informed consent, approved by the Miriam Hospital IRB Review Board, was provided by all study participants. In this study, BV was diagnosed by the same expert gynecologist (SC) using Amsel's criteria. BV was considered to be present if any 3 of the following 4 conditions were noted: abnormal discharge, vaginal pH > 4.7, the presence of clue cells on a wet mount of vaginal secretions, or a positive “whiff test.” A smear from the posterior fornix was examined under oil immersion (x1000) to determine the vaginal white cell count and the percentages of polymorphonuclear lymphocytes (PMNs) and monocytes.

**CVL specimens**

CVL specimens were obtained by instilling 10 ml of sterile saline into the vaginal vault directing the stream of fluid at the cervical os. The fluid was left for about 30 seconds and then aspirated. The fluid was then transferred to a sterile 15 ml conical test tube and stored at −70°C.

**Cytokine assays**

Frozen CVL samples were thawed and centrifuged at 500x g to remove debris; they were then centrifuged at 2000x g and supernatants were harvested. The resulting supernatant was divided in two aliquots, and cytokine concentrations were measured using commercial quantitative sandwich enzyme immunoassay kits (Quantikine, R & D Systems, Minneapolis, MN). All samples were run in duplicate and results were reported in pg/ml. Samples were diluted 1 : 10 for IL-1β and IL-8 and 1 : 4 for IL-6 according to manufacturer's directions. Assays yielding values below the limit of detection were left-censored at the lower limit of detection, corresponding to 0.7 pg/ml for IL-6, 10 pg/ml for IL-8, and 1.0 pg/ml for IL-1β.

**Data analysis**

Sample medians and ranges were determined. Estimates of the cumulative distribution function (CDF) were plotted by BV status for each cytokine. Since genital tract cytokines have complex distributions, CDF plots of cytokine concentrations for patients at consecutive visits, with and without BV, provided the most comprehensive presentation of these data [15]. A stratified Cox proportional hazards model was used to compare the distribution of cytokine levels by BV status, and by participant characteristics. The association of IL-8 and BV was examined using a multivariate linear regression model. Generalized estimating equations (GEE) were used since repeated measures were taken from specimens from the same participants. Statistical analyses were performed using SAS version 8.2 (SAS, Cary, NC) and R (www.r-project.org).

## 3. RESULTS

**Participants**

Longitudinal CVL samples from 24 women who were HERS participants in Providence, Rhode Island were selected based on their BV status at sequential visits; 16 of the women selected were HIV-infected and 8 were high-risk HIV-uninfected. The mean age of these women was 31 years (range 24.0–40.7); 16.7% were African-American, 20.8% were Latin, and 62.5% were Caucasians. At baseline, the median log^10^ plasma HIV RNA concentration of HIV-infected women was 3.04 (range: undetectable to 4.88), 31% (5/16) had undetectable plasma HIV RNA concentrations and 25% had CD4^+^T cell counts <200 cells/*μ*l. Sixty eight percent (11/16) of the HIV-infected women were on antiretroviral regimens at the time of study entry. All participants were prescribed oral metronidazole 500 mg to be taken twice daily for seven days when BV was diagnosed.

**Cytokine detection**

IL-6, IL-8 and IL-1β concentrations in CVL samples were evaluated from women who were selected for this study. Concentrations of each of these cytokines in genital fluids in the presence and absence of BV were compared since BV is associated with greater susceptibility to HIV and it was suspected that BV might change the cytokine environment in the genital tract. Of the 48 paired specimens from the 24 participants, IL-8 was detected in 99%, IL-1β in 83%, and IL-6 was detected in 56% of all CVL samples.

**Associations between cytokines and BV status**

**Figure 1:**
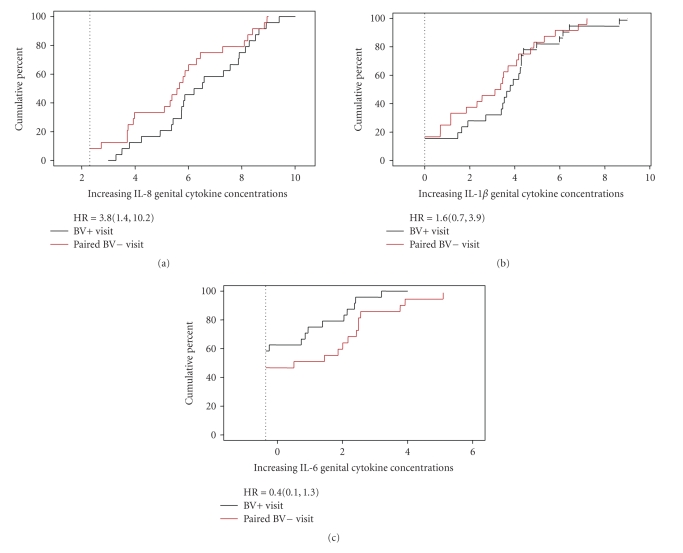
Plots of the estimated cumulative distribution function (CDF) curves for each cytokine by BV status, BV positive (BV+) or BV negative (BV−), in pg/ml (log^10^), as well as the hazard ratios and corresponding 95% confidence intervals estimated using a stratified Cox proportional hazard model. IL-8 concentrations when women were BV+ (red line) were significantly higher compared to IL-8 concentrations (black line) after the women cleared BV (Hazard ratio = 3.8, 95% CI 1.419, 10.177). The differences in genital tract IL1-β and IL-6 concentrations when women were BV+ versus BV− were not statistically significant.

Genital tract IL-8 concentrations were significantly higher when women were diagnosed with BV than after the same women had cleared BV (Hazard ratio = 3.8, 95% CI 1.419, 10.177). In contrast, there was no significant difference in genital tract concentrations of IL-1β or IL-6 associated with BV clearance (Figure 1). Trends in overall cytokine concentrations were similar in both HIV-infected and uninfected participants; no statistically significant interactions between HIV and BV status were observed Table 1. Among the HIV-infected women, there was no significant difference in vaginal IL-8 concentrations by BV status between women who were on antiretroviral therapy compared to those who took no antiretroviral drugs during the study (data not shown).

**Adjusted association between BV and IL-8**

The within-subject changes in genital tract IL-8 concentration associated with BV status were evaluated by means of a multivariate linear regression. After adjusting for the effects of ethnicity, HIV status, quantity of vaginal PMNs and monocytes, and genital tract IL-1β concentrations, the association between BV and increased genital tract IL-8 remained significant. The transition from having BV to documented clearance of BV was associated with a 2.4 fold decline in genital tract IL-8 concentration that was statistically significant (p=0.02) and independent of the effect of the other factors controlled for in the regression model.

**Cytokine levels associated with incident BV and BV clearance**

**Figure 2:**
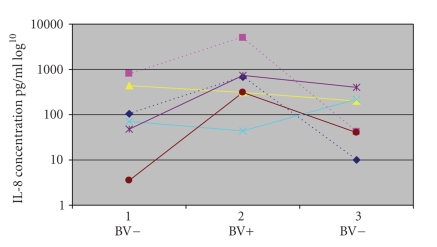
Genital tract IL-8 concentrations in pg/ml (log^10^) were evaluated at three consecutive visits for 2 high-risk HIV uninfected (dotted lines) and 4 HIV infected women (solid lines) At baseline, visit 1, none of these women had bacterial vaginosis. Women were only included if data for all 3 visits was available.

For 7 women, 5 HIV-infected and 2 high-risk HIV-uninfected, genital cytokine concentrations were evaluated at three consecutive clinic visits each six months apart. These women were BV negative at their initial visit, then they developed BV and were diagnosed at their second visit. Treatment was initiated and they were BV negative at their next visit. Participants included in this analysis had no other concomitant genital tract infection or other contraindication for CVL collection at any of the three visits. Median concentrations and ranges of genital tract IL-1β and IL-8 at baseline, before BV diagnosis, at the time of BV diagnosis, and after BV clearance are shown in Table 2. A GEE model comparing repeated measures in the same women demonstrated a significant increase in mean genital tract IL-8 concentrations but not IL-1β when women were diagnosed with BV compared to concentration of these cytokines when they did not have BV, p=.004 and p=.51, respectively (Figure 2). IL-6 concentrations could not be evaluated as over 70% of the observations were below the level of detection of our assay.

## 4. DISCUSSION

We found that BV is associated with a significant increase of IL-8 concentration in the female genital tract of HIV-infected and high-risk uninfected women, unrelated to IL-1β or IL-6 activity. One cross-sectional study found no difference in IL-8 levels in HIV-uninfected women with BV compared to healthy controls, which is at variance with the current study [12]. This may be explained because genital IL-8 concentrations in healthy women are highly variable and, therefore, may be difficult to interpret in cross-sectional studies, unlike the current design in which sequential specimens from the same women were compared. A study evaluating genital tract cytokine expression in HIV-uninfected pregnant women before and after BV treatment, in which each woman served as her own control, found significant decreases in genital tract IL-8 levels for women who cleared BV, as observed in the current study [11]. Other studies have found no increase in IL-6 associated with BV [16], as was observed in the current study.

Lower IL-1β concentrations after BV clearance were observed in the current study, which is consistent with studies that have suggested women with BV have higher genital tract IL-1β levels than healthy controls. Changes in IL1-β concentrations, in women with and without BV, were not statistically significant. This may be attributable in part to the fact that the current study evaluated HIV infected and high-risk uninfected women whose baseline genital tract IL-1β may have already been elevated compared to low-risk seronegative women, and thus subtle changes may have been missed. Although IL-1β is a potent inducer of IL-8, we observed higher concentrations of IL-8 when women had BV compared to levels after clearing BV, even after adjusting for IL-1β in the multivariate analysis. Expression of IL-8 can also be induced by IL-1β-independent mechanisms in cervical and vaginal epithelial cells directly by microorganisms [17]. The current study's findings are consistent with observations of Zhang et al. that infection with *Mycoplasma hominis* alters cytokine gene expression in cervical epithelial cells, resulting in upregulation of IL-8 [18]. The lack of significant change in IL-1β or IL-6, despite significant increase in IL-8 observed in this cohort, may be attributable to suppressive effects of specific BV-associated organisms on the local mucosal immune environment [5]. Further studies to identify whether some of the more common organisms associated with BV, such as Gardnerella or Mobiluncus or other more recently characterized organisms are most responsible for the up regulation of genital tract IL-8, are warranted [19].

The observed trends in genital tract cytokine concentrations were similar for HIV-infected and high-risk HIV-negative women. The majority of HIV-infected women in this study were on antiretroviral medications and had CD4 T cell counts > 200 cells/*μ*l and thus tended to be immunocompetent. Others have noted that immunocompromised HIV-infected patients have significantly elevated concentrations of IL-8 in plasma and genital tract secretions [20]. By decreasing vaginal IL-8 concentrations, the treatment for BV could potentially decrease the HIV RNA levels in this compartment, and thereby, decrease the risk of HIV transmission. Similarly, in high-risk HIV-uninfected women, BV treatment may decrease susceptibility to HIV infection by reducing IL-8 concentrations [8].

Though there were relatively few women included in this study, the comparison of vaginal cytokine concentrations from the same individual with and without BV eliminated individual variability. Moreover the use of archived samples, known to be free of sexually transmitted infections that can affect cytokine levels in the vaginal tract, strengthened our ability to evaluate the association between BV and vaginal cytokine level. Future studies should include larger numbers of HIV-infected women, thus enable a more thorough analysis of the possible effect of antiretroviral therapy on vaginal cytokine concentration.

In the light of the high prevalence of BV among HIV-infected women and women at risk for HIV infection and the association of BV with increased genital tract HIV RNA levels, understanding the impact of BV on the immune environment of the genital tract may facilitate effective HIV prevention. Interventions that are able to impede IL-8 production and function may reduce genital tract HIV viral load and impact transmission and acquisition of HIV. Interventions that treat and prevent BV may also have an impact on HIV transmission and prevention.

## Figures and Tables

**Table 1:** Median values and ranges of genital tract IL-8, IL-1β, and IL-6 concentrations (pg/ml) by bacterial vaginosis (BV) status, stratified by HIV status.

Cytokine	BV status (+/−)	HIV+ median cytokine concentration (range) pg/ml	HIV− median cytokine concentration (range) pg/ml
IL-8	BV+	609.9 (33.3–12071.9)	508.0 (26.7–4987.6)
	BV−	475.3 (40.7–7659.4)	46.4 (10–329.2)
IL-1β	BV+	58.1 (1–5665.4)	18.3 (1–396.8)
	BV−	36.2 (< 0 1346.5)	1.9 (1–127.9)
IL-6	BV+	0.7 (0.7–24.5)	0.7 (0.7–2.6)
	BV−	7.44 (0.7–162.7)	0.7 (0.7–11.3)

**Table 2:** Median genital tract concentrations of IL-8, IL-1β, and IL-6 (pg/ml) at 3 consecutive clinic visits 6 months apart.

Cytokine	Visit 1 BV−	Visit 2 BV+	Visit 3 BV−
IL-1β	28.5 (13.9–50.8)	15.1 (1–396.8)	3.2 (1–127.7)
IL-8	86.9 (3.5–791.8)	680.7 (44.1–4967.6)	202.8 (9.9–405.6)
IL-6 ^1^	0.7 (0.7–5.5)	0.7 (0.7–2.3)	0.7 (0.7–4.2)

^1^>70% of IL-6 concentrations were below the level of detection of the assay.
